# Spongiform leukoencephalopathy unveiled in an autopsy of a drug abuser

**DOI:** 10.4322/acr.2023.465

**Published:** 2024-01-08

**Authors:** Hemlata Jangir, Govinda Balmuchu, Jhansi Lakshmi Mylapalli, Arulselvi Subramanian, Sanjeev Lalwani

**Affiliations:** 1 All India Institute of Medical Sciences, Jai Prakash Narayan Apex Trauma Centre, Department of Laboratory Medicine, New Delhi, India; 2 All India Institute of Medical Sciences, Jai Prakash Narayan Apex Trauma Centre, Department of Forensic Pathology and Molecular DNA, New Delhi, India

**Keywords:** Forensic Toxicology, Leukoencephalopathy, Progressive Multifocal, Psychoses, Substance-Induced

## Abstract

Toxic leukoencephalopathy (TLE) is a rare neurological debilitating and fatal condition. It has been previously associated with exposure to leukotoxic offenders such as chemotherapy, cranial radiation, certain drugs, and environmental factors. Currently, it is a commoner white matter syndrome resulting from increased substance abuse, classically by inhaled heroin and other opioids. Herein, we report a case of fatal TLE unveiled in an autopsy of a drug abuser. A 24-year-old male was found dead on the roadside. A day before, he was located in a state of delirium. In this case, the autopsy findings and histopathology characteristics of cerebral cortex involvement particularly directed to speculate the heroine as the principal offender.

## INTRODUCTION

Toxic leukoencephalopathy is a rare, rapidly progressive but potentially debilitating and fatal disorder affecting the brain white matter, particularly the myelin sheath. It has been previously reported in association with exposure to various leukotoxic offenders such as chemotherapy, cranial radiation, certain drugs, and environmental factors. In the current era, such toxic-induced cerebral white matter degenerative findings are rising due to growing illicit substance abuse. Particularly, inhalation of heroin vapor results in distinct imaging and brain biopsy features of white matter leukoencephalopathy.^1-3^ Similar findings have been observed in other opioids and cocaine abuse.^4^ The clinical course varies from an unexpectedly favorable outcome in some cases and rapid progression to death in others.^1^ We report a rare case of fatal TLE unveiled in an autopsy of a 24-year-old man with a history of unknown substance abuse. This case was directed to speculate the heroine as the major offender, relying heavily on the clinical autopsy details and characteristic histopathological pattern of the brain involvement.

## CASE REPORT

A 24-year-old male, previously enrolled in a rehabilitation program, was found dead and subsequently brought for autopsy. The patient was reported to be in a state of delirium a day before his death. He had a history of drug abuse, although the specific drug was unknown. The autopsy was performed after 23 hours of the death. During the autopsy, the brain parenchyma showed greenish discoloration and spongiform changes, particularly in the frontal cerebral white parenchyma. The sample was collected from the part of the affected brain and was received for histopathological evaluation.

## HISTOPATHOLOGY

**Figure 1:**
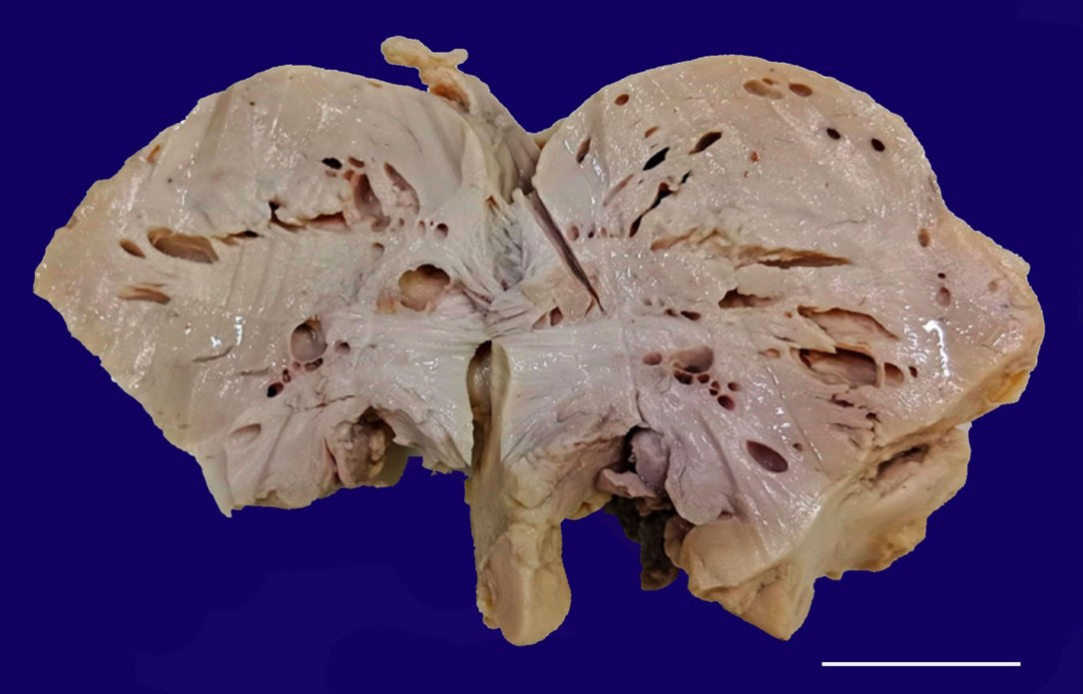
Gross specimen revealing marked cortical atrophy with spongiform appearance of white matter predominantly (scale bar = 5 cm).

Gross examination of the brain Parenchyma showed marked atrophic changes with loss of gyri and sulci. Multiple cystic cavities were present predominantly in the white matter (Figure 1).

**Figure 2:**
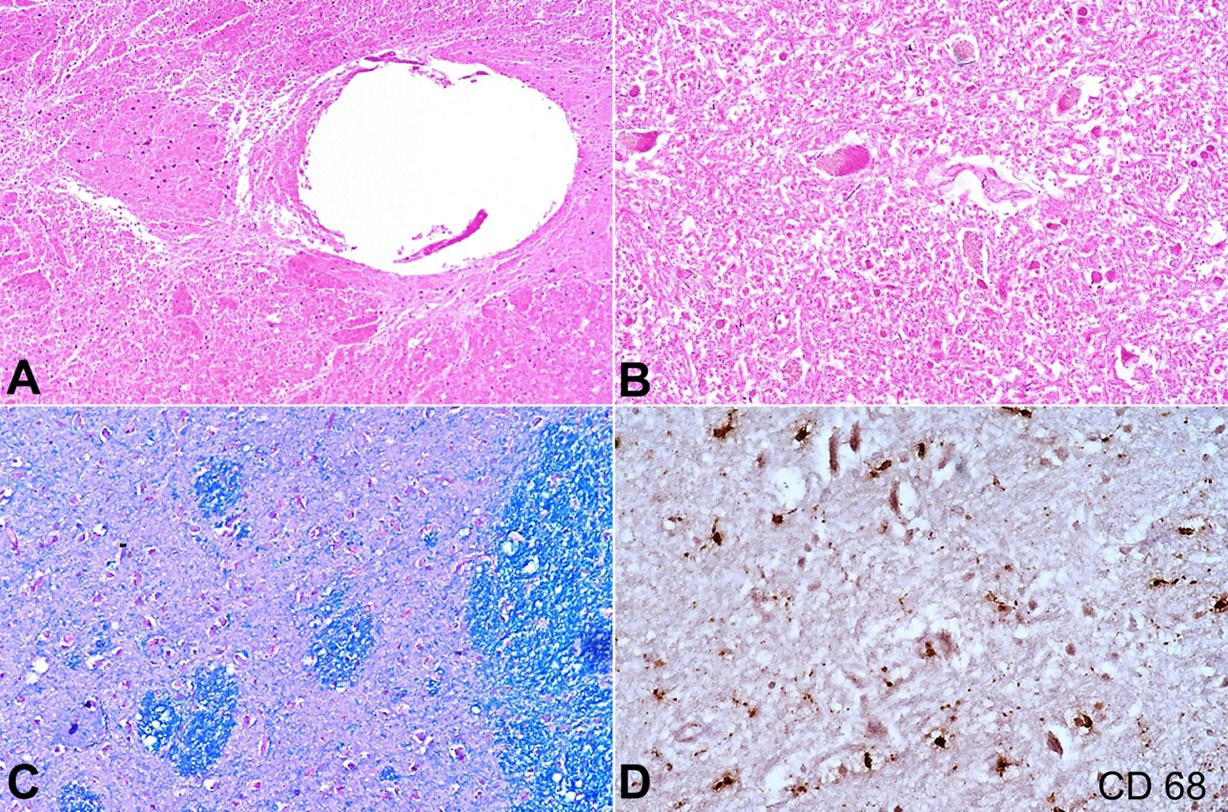
Photomicrographs of the central nervous system. **A –** demonstrates spongiform/vacuolar degeneration of the white matter in a background of reactive gliosis and infarct necrotic areas (H&E, 100x); **B –** shows numerous axonal spheroids, and macrophages infiltration in the degenerated cystic parenchyma (H&E, 400x); **C –** Luxol-Fast Blue (LFB) stain revealing rarefaction of myelinated nerve fibers *(200x)*; **D –** CD68 IHC stain highlighting the macrophages (400x).

The microscopic evaluation revealed extensive spongiform or vacuolar degeneration in the form of pseudocyst formation of the deep white matter, axonal swelling, and axonal loss (Figure 2A). Numerous axonal spheroid bodies were present, along with occasional calcospherites. Multiple foci of parenchymal infarction and edema were seen. Oligodendrocytes showed clustering of nuclei. Macrophage infiltration was seen. Blood vessels were normal, and there was no evidence of any inflammatory or infective process (Figure 2B). Myelin stain revealed diffuse demyelination and axonal loss (Figure 2C). CD 68 immunohistochemistry (IHC) highlighted the infiltration of macrophages in the parenchyma (Figure 2D).

Histological features favored the autopsy finding consistent with toxic leukoencephalopathy, likely induced by heroin as a major offender.

## DISCUSSION

The report of leukoencephalopathy in association with heroin inhalation was described as a newer entity by Wolters et al.^1^ in 1982.^1^ It is a well-documented condition in medical literature, associated with various known offenders such as chemotherapy, cranial radiation, certain drugs, and environmental factors.^5^ In today's era, the use of substances or abusing drugs is the rising offender for the increasing TLE cases, classically by inhaled heroin and also by other opioids and cocaine.^4,6^

However, the act of inhaling the smoke resulting from the heroine powder after burning over the aluminum foil was previously thought to be the only major culprit leading to the spongiform degeneration of white matter. However, newer evidence suggests that injecting and snorting as other modes of administration are also likely to express the changes related to heroin-induced TLE.^7^

Despite numerous causes of leukoencephalopathy, the specific term “spongiform” vacuolization in this condition is unique to toxic etiologies.^5^ However, the precise pathophysiology and predisposing factors for drug-induced leukoencephalopathy are not fully understood. Current theories are primarily based on post-mortem tissue studies. These theories suggest that axonal injury may be the initial damage, followed by subsequent demyelination as a secondary process.^8^ One prevailing hypothesis suggests that the spongiform pattern seen in leukoencephalopathy resembles an incomplete infarct pattern, which could be linked to partial hypoxic injury, often observed in cases of multiple substance abuse. The white matter tracts are particularly vulnerable to such injuries due to their high metabolic demands and extensive nature.^6^ Some believe that the lipophilicity of many toxic compounds enables their entry into the lipid-rich brain, which is particularly relevant to the growing problem which is particularly relevant to the growing problem of substance abuse.^9^

In addition to hypoxic injury, impairing mitochondrial function is also believed to play a role in the development of spongiform white matter changes in certain cases of leukoencephalopathy.^10^ Notably, two cases of leukoencephalopathy associated with the inhalation of heroin vapors have indicated a potential defect in mitochondrial function, as evidenced by elevated white matter lactate levels. Interestingly, these cases have shown a favorable response to antioxidant treatments.^11^ This suggests that a defect in mitochondrial function may contribute to the pathophysiology of drug-induced leukoencephalopathy in some instances, highlighting the complexity of its underlying mechanisms.

As the heroin pyrosylate is often prepared on aluminum foil, the possibility of concurrent aluminum leukoencephalopathy exists, but the symptoms of isolated aluminum leukoencephalopathy are found to be strikingly different from heroin leukoencephalopathy.^12^

The onset of symptoms is also highly variable, with declines occurring anywhere from hours to months after exposure, with an average latency period of approximately three weeks reported in some studies.^13^

Toxic leukoencephalopathy presents a broad spectrum of clinical symptoms varying significantly from mild manifestations such as restlessness, bradyphrenia, and apathy to severe neurobehavioral consequences manifestations like abulia, akinetic mutism, stupor, coma, and even fatality.^1,14^ Furthermore, the impact of toxic white matter injury can have lasting neurobehavioral implications long after the initial exposure. It is believed that the toxin can accumulate in fatty myelin, gradually causing ongoing tissue damage and symptom development.^6^

There can be a delay between exposure to toxins and the appearance of symptoms by a phenomenon known as “coasting.” This delay might explain negative toxicology results in some cases. Alternatively, these can also be undetectable in the bloodstream when symptoms finally manifest, as the toxin substances could trigger a persistent metabolic change even after discontinuation. In our case, the toxicologic laboratory work-up could not be performed, which was limited to reflect the exact substance abused.^15^

The neuroimaging findings suggest that heroin inhalation toxicity typically results in diffuse symmetrical white matter involvement, with a notable sparing of the subcortical U fibers, particularly targeting the cerebral hemispheres. Notably, this condition is distinguished by the absence of cerebellar and brain stem involvement. However, in cases of impure heroin use, there may be additional involvement of the cerebellar and posterior cerebral white matter and the posterior limb of the internal capsule. Furthermore, compared to other forms of heroin toxicity, it tends to have a frontal rather than occipital predominance and involves more prominent axonal damage.^16^ Similar striking features are noted in this case.

Histopathology remains the gold standard for the confirmative diagnosis. TLE has peculiar cerebral white matter degeneration, including marked spongiform or vacuolar degeneration. Affected parenchyma shows widespread myelin pallor with reactive gliosis, numerous axonal spheroid bodies, macrophage infiltration, edematous, multifocal infarct areas, and long tract degeneration. Demyelination and marked loss of axon are the hallmarks of TLE.^17,18^ Normal blood vessels and the absence of inflammation are also features of this entity. In the current case, the Luxol-Fast Blue (LFB) stain demonstrated the extension loss of the myelin sheath of the axons. Electron microscopy revealed vacuole formation in oligodendroglia and myelin sheaths with deep white matter edema and abnormal mitochondria.^11^ It is distinguishable from hypoxia-induced encephalopathy by the absence of intra-myelin vacuole formation.^19^

Though this disease lacks a specific treatment, antioxidant therapy holds therapeutic potential. In resource-limited settings, turmeric extract, whose neuroprotective and antioxidant roles are well studied, may have a role in treatment.^20^

## CONCLUSION

Diagnosing such rare entities relies on recognizing the histo-morphological clues, which can be key to pursuing the appropriate diagnostic support.

Awareness of such neurological sequelae may help physicians to make patients aware of dangerous neurological outcomes resulting from drug abuse.

Although the diagnosis of toxin-induced leukoencephalopathy is rare, it is important to consider in every unknown autopsy without any obvious cause of death.
